# A Market Survey of Pesticide Residues in Fruit Juices in Slovenia in 2026

**DOI:** 10.3390/foods15193521

**Published:** 2026-10-01

**Authors:** Helena Baša Česnik

**Affiliations:** Central Laboratories, Agricultural Institute of Slovenia, Hacquetova ulica 17, SI-1000 Ljubljana, Slovenia; helena.basa-cesnik@kis.si or helena.basa@kis.si

**Keywords:** pesticide residues, PFAS, juice, consumer safety

## Abstract

Although the European Food Safety Authority (EFSA) encourages the monitoring of pesticide residues in food, only 8.4% of processed fruit samples were monitored in the EU in 2023. In addition, the literature mainly focuses on pesticide residues in fruit rather than in juices. In the present study, 91 fruit juice samples were analyzed using GC-MS/MS for the presence of 32 active substances. The validated analytical method used was compliant with SANTE/11312/2021. Of the samples inspected, 29.7% were positive for at least one of the 32 targeted substances, while 6.6%, or 1.1, after calculation refinement by an alternative scenario, potentially exceeded the Maximum Residue Level (MRL). Potential MRL exceedances of fluopyram in table grape and mango, kresoxim-methyl in apple, tebuconazole in mango, and trifloxystrobin in apple did not pose a chronic or acute risk to consumers. Acute risk to consumers was theoretically calculated for apples containing tebuconazole. After calculation refinement by an alternative scenario, no acute risk was theoretically observed.

## 1. Introduction

In the European Union (EU), a reduction in the sale of plant protection products (PPPs) has been observed, with the latest data showing that, in 2023, approximately 292,000 tons of pesticides were sold in the EU, which is 9% less than in 2022. However, in Slovenia, the Statistical Office has reported that around 738 tons of PPPs were sold in 2024, which is 3% higher than in 2023. These data demonstrate that the amounts of PPPs used by farmers remain high [1,2].

The exposure of human populations to pesticides can lead to hematological and neurological disorders [3], potentially causing cancer [4], diabetes [5], central nervous system disorders (Parkinson’s disease, Alzheimer’s disease) [6], reproductive system disorders [7,8], endocrine disruption [9], and cognitive impairment among older people [10].

To protect consumers, annual monitoring of pesticide residues in food is conducted in the EU to ensure that food on the market is safe. MRL exceedances were observed in the EU from 2020 to 2023 in 3.7% to 5.1% of fruit, vegetables, cereals, and animal-origin food samples [11,12,13,14], representing a potential risk to consumers.

In Slovenian studies, fruit was found to contain more active substances than vegetables [15], and the proportion of fruit samples that tested positive for pesticide residues (74.2%) was higher than that of vegetables (21.0%) [16]. In the latest research, 59.1% of analyzed fruit in Slovenia tested positive, with concentrations up to 0.13 mg/kg [17]. A similar percentage of positive fruit samples (52.9%) was found in Turkey, with the highest concentration of 1.6 mg/kg [18], and in Saudi Arabia (50.0%), with the highest concentration of 2.9 mg/kg [19]. The first study in Poland, conducted in 2024, found a slightly higher percentage of positive samples (65.8%), with concentrations reaching up to 7.1 mg/kg [20], while a second study in Poland in 2026 found much higher percentages, with 91% of fruit samples containing pesticide residues at concentration levels up to 2.2 mg/kg [21]. A similar observation was made in the first studies in China [22] and Egypt [23], where 88.6% and 88.1% of fruit samples contained pesticide residues, respectively. In contrast, in Jordan and in the second study in China, positive fruit samples represented only 26.4% and 24.5% of inspected samples, with the highest concentrations being 3.7 mg/kg and 3.4 mg/kg, respectively [24,25].

Based on the high percentage of positive fruit samples worldwide, pesticide residues are also expected to be found in processed fruit products such as fruit juices. However, in 2023, only 8.4% of inspected samples monitored in the EU were processed fruit [14], and there are fewer investigations of pesticide residues in juice compared to fruit. From 2011 to 2026, at least eight surveys were conducted in which pesticide residues were measured in fruit juice [26,27,28,29,30,31,32,33]. Among other compounds, azoxystrobin, boscalid, cyprodinil, fludioxonil, iprovalicarb, clomazone, kresoxim-methyl, metazachlor, myclobutanil, pendimethalin, penconazole, pyraclostrobin, pyrimethanil, pirimicarb, pyriproxyfen, tebufenpyrad, tebuconazole, tefluthrin, tetraconazole, and trifloxystrobin were analyzed in these surveys, as in the present study. Pesticide residues not only reduce the quality of fruit juice but can also alter its flavor and produce a peculiar smell, representing another reason for monitoring pesticide residues in fruit juices [33].

In previous studies, pomegranate [32], grape [26,28,30], guava [30], mango [26,30], apple [26,28,29], orange [26,28,30], peach [26,28], strawberry [26,27,28,31], pear [26,28], raspberry [27,28], cherry [28], apricot [28], blueberry [28], plum [28], pineapple [26,28], currant [27], mandarin [26], banana [26], and grapefruit [26] juices were analyzed. However, only one study [26] included seven multifruit juices. To the best of our knowledge, apart from our study in 2025 [34] and a study conducted in 2011 [26], the present study is among the few in which fruit juices produced from two or more types of fruit were studied alongside fruit juices produced from one type of fruit. As in the 2025 study [34], the fruit composition of the juices is reported in the present study.

This survey focuses on pesticide residues in fruit juices on the Slovenian market in 2026, produced in various countries. The active substances authorized in Slovenia and the EU were reviewed in 2022 [35,36], and thirty-two that are amenable to gas chromatography and have been successfully validated were selected. Eleven of these active substances are fluorinated pesticides, all of which are, according to the new OECD definition, PFASs (per- and polyfluoroalkyl substances), as they contain at least one fully fluorinated methyl or methylene carbon atom (substances containing a CF_3_ or CF_2_ (=CF_2_ or –CF_2_–) group, without H, Cl, Br, or I). PFASs are persistent and toxic compounds that, in recent years, have been of great interest in food safety. Among the thirty-two active substances, six were insecticides/acaricides, eight were herbicides, and eighteen were fungicides. Measured concentrations in fruit juices were recalculated to estimate the concentrations in fruit, while compliance with MRLs in fruit was evaluated and a consumer risk assessment was conducted.

The present survey, conducted in 2026, is a continuation of a previous survey carried out in 2025 in which only 40 fruit and vegetable juices were analyzed [34]. The current study includes over twice as many samples as the previous one (some were sampled both years), with a larger proportion of juices originating from countries outside of Slovenia (37.4%, compared with 12.5% in the previous study). Juices in the 2025 study were collected and analyzed to demonstrate the robustness of the analytical method presented in previous work [34]. The present study focuses on pesticide residues in juices in the context of food safety monitoring.

## 2. Materials and Methods

### 2.1. Sampling

Ninety-one samples of fruit juices were collected from wholesaler Slovenian stores in March 2026: seventy-four were packed in 1 L packages and seventeen in 0.2 L packages. Twenty-four samples were produced in Austria, two in Croatia, one in Hungary, five in Italy, two in Serbia, and fifty-seven in Slovenia. No sample was designated as organic. A description of the samples is presented in Table 1.

All samples were randomly selected to cover all types of juices from specific producers: Dana, Smodin, Presad, Moj dan, Fructal, and Lumpi from Slovenia, and Rauch, Vindi, Lumpi, S-Budget, Spar, and Despar from other countries. It should be noted that relatively few juices from other countries are sold in Slovenia, as there are many domestic producers.

For each type of juice, one sample was collected. Four samples from Fructal packed in 0.2 L containers had the same label as four samples packed in 1 L containers.

Since samples were collected within one month, seasonal variability is not addressed. The present study is also limited by its unequal and non-representative sampling of foreign countries, where some countries are represented by only one or two samples.

Thirteen samples, 26-593, 26-596, 26-597, 26-609, 26-610, 26-715, 26-718, 26-726, 26-729, 26-738, 26-744, 26-745 and 26-746, were assessed by type of juice and producer in 2026, overlapping with samples from 2025.

### 2.2. Analyses

#### 2.2.1. Chemicals

The certified standards were obtained from Dr. Ehrenstorfer (Augsburg, Germany), while acetone p.a., acetone HPLC-grade, dichloromethane p.a., and petroleum ether p.a. were supplied by J.T. Baker (Deventer, The Netherlands). All other chemicals were from Sigma-Aldrich (Steinheim, Germany).

#### 2.2.2. Extraction and Determination

The samples were buffered with sodium acetate and acetic acid, and then extracted with a mixture of acetone, dichloromethane, and petroleum ether at a 1:2:2 (*v*:*v*:*v*) ratio. Determination was performed using a gas chromatograph (Agilent Technologies 8890, Shanghai, China) coupled with a tandem mass spectrometer (Agilent Technologies 7010B, Santa Clara, CA, USA), equipped with a Gerstel 20PRE0795 multipurpose sampler (Gerstel, Sursee, Switzerland). More detailed extraction and determination procedures have been published elsewhere [34].

The studied active substances were azoxystrobin, benthiavalicarb-isopropyl, boscalid, clomazone, cyprodinil, flonicamid, fluazifop-p-butyl, fludioxonil, flufenacet, fluopicolide, fluopyram, flutolanil, iprovalicarb, kresoxim-methyl, lambda-cyhalothrin, metazachlor, metribuzin, myclobutanil, napropamide, penconazole, pendimethalin, pirimicarb, proquinazid, prosulfocarb, pyraclostrobin, pyrimethanil, pyriproxyfen, tebuconazole, tebufenpyrad, tefluthrin, tetraconazole, and trifloxystrobin. Eleven of these are classified as PFASs: flonicamid, fluazifop-p-butyl, fludioxonil, flufenacet, fluopicolide, fluopyram, flutolanil, lambda-cyhalothrin, tefluthrin, tetraconazole, and trifloxystrobin.

Calibration was performed using matrix-matched standards in apple juice.

#### 2.2.3. Validation Parameters for Fruit Juice

The limit of quantification (LOQ) for all thirty-two active substances was 0.005 mg/L. Linearity ranged from 0.005 to 0.03 mg/L or 0.005 to 0.04 mg/L, with R^2^ ≥ 0.99. Recoveries obtained during validation ranged from 72.2% to 104.2%, with RSDs from 6.3% to 18.0%. Measurement uncertainties of repeatability ranged from 7.0% to 26.0%, and of reproducibility from 12.7% to 36.0%. The method complies with SANTE 11312/2021 [37], and more detailed validation parameters have been published elsewhere [34]. In the Supplementary Materials, LOQs, procedural recoveries, RSDs, and matrix effects are reported. Average procedural recoveries obtained during the analyses were in the range 74.9–100.7%, with RSDs of 2.3–16.6%. The matrix effect was 38–363%.

### 2.3. Calculation of Residues in Fruit

Pesticide residues in juices were divided by fruit portion and processing factors (PFs) from the EU database [38], if available, to determine pesticide residues in fruit. PFs from the EU database are presented in the Supplementary Materials. Where PFs were not available, a PF of 1 was used. Each PF is the only one reported in the EU database [38] for a specific combination of active substance and juice type. If PFs for grape juice were, for instance, used for mango juice, a significant error could occur in the calculations.

A density of 1 kg/L was used to recalculate the estimated concentration in fruit from juice.

The formula for calculating the residues in fruit from residues in juice was

C (fruit) in mg/kg = C (juice) in mg/L × 100% / (fruit portion in % × PF).

As a worst-case scenario, it was assumed that all residues in the juice originated from each fruit ingredient. The calculated pesticide residues in fruit were compared with MRLs and used for consumer risk assessment.

For fruit exceeding MRLs, a refinement of concentration in fruit was conducted less conservatively using the whole fruit portion as an alternative scenario:

C (fruit) in mg/kg = C (juice) in mg/L × 100% / (sum of fruit portion in % × PF)

The sum of the fruit portion is the sum of the individual fruit portions for each type of fruit in the juice.

### 2.4. Consumer Risk Assessment

Consumer risk assessment was conducted using the European Food Safety Authority (EFSA) PRIMO model Rev. 3.1. This model includes more than thirty EU diets but does not include processed food. Input values for chronic exposure were the median values of pesticide residues in fruit and Acceptable Daily Intakes (ADIs). The model calculates chronic exposure by multiplying median residues by mean consumption data at the 50th percentile (the latter is already included in the model). The resulting value is divided by the ADI and multiplied by 100 to express exposure as a percentage of the ADI. Input values for acute exposure were the maximum values of pesticide residues in fruit and Acute Reference Doses (ARfDs). The model calculates acute exposure by multiplying the highest residues by large-portion consumption data at the 97.5th percentile (the latter is already included in the model). The resulting value is divided by the ARfD and multiplied by 100 to express exposure as a percentage of the ARfD. Where an ARfD was not allocated, the ADI was used instead as a worst-case scenario. The ARfD value is always set higher than or equal to the ADI. Acceptable chronic exposure is ≤100% of the ADI, and acceptable acute exposure is ≤100% of the ARfD.

### 2.5. Data

In this paper, only results at or above the LOQ are reported, as suggested by the French Accreditation Committee COFRAC, when the range of pesticide residue analyses was accredited according to SIST EN ISO/IEC 17025 [39]. The laboratory itself revoked accreditation in the field of pesticide residues because the financial costs outweighed the benefits.

The median was calculated from the positive results with residues at or above the LOQ. For consumer exposure, only residues at or above the LOQ were used.

## 3. Results and Discussion

### 3.1. Measured Residues in Juice

In the present study, positive juice samples were defined as those in which pesticide residues (from the list of thirty-two analyzed active substances) were found at or above the LOQ. Negative juice samples were defined as those in which pesticide residues (from the list of thirty-two analyzed active substances) were not found, as they were either absent or below the LOQ.

**Figure 1:**
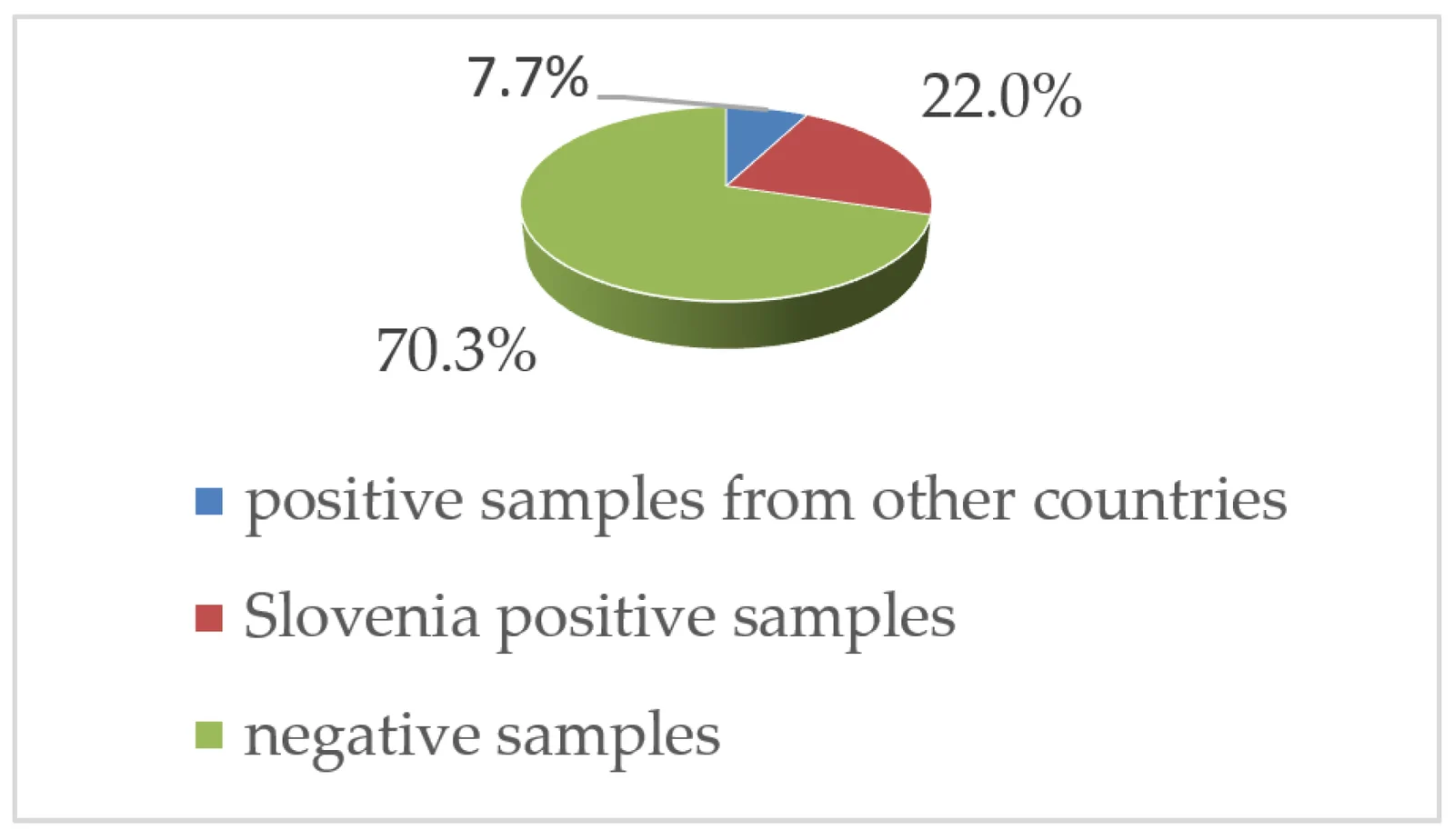
Proportions (%) of positive samples and negative samples by country across all 91 samples.

In the present research, twenty-seven fruit juice samples were positive for at least one of the thirty-two targeted substances (29.7%): five from Austria (20.8% of Austrian samples), two from Italy (40.0% of Italian samples), and twenty from Slovenia (35.1% of Slovenian samples). In addition, 20.6% of samples from foreign countries were positive for at least 1 of the 32 targeted substances (7 out of 34 samples). When comparing positive samples with all samples analyzed from each country using Fisher’s exact test, the *p* value was 0.445 for Austria–Slovenia, 1.00 for Italy–Slovenia and 0.602 for Austria–Italy. Since *p* > 0.05, the differences in proportions are not statistically significant. The results are presented in Figure 1.

Regarding packaging, 3 out of 17 juice samples packed in 0.2 L vessels (17.6%) were positive, while 24 of 74 juices packed in 1 L vessels (32.4%) were positive. When comparing positive samples with all samples analyzed for both types of packaging using Fisher’s exact test, the *p* value was 0.559. Since *p* > 0.05, the differences in proportions are not statistically significant. All three positive samples packed in 0.2 L vessels originated from Slovenia.

Eleven juice samples contained one active substance, eight samples contained two active substances, four samples contained three active substances, three samples contained four active substances, and one sample contained five active substances. The proportion of juice samples with multiple residues was 17.6% (16 out of 91 samples): 5 from other countries (14.7% of samples from other countries) and 11 from Slovenia (19.3% of Slovenian samples). Regarding packaging, 3 out of 17 juice samples packed in 0.2 L vessels (17.6%) (all positive 0.2 L samples) and 13 out of 74 juice samples packed in 1 L vessels (17.6%) contained multiple residues. When comparing positive samples with multiple residues with all samples from Slovenia and abroad using Fisher’s exact test, the *p* value was 0.781. Since *p* > 0.05, the differences in proportions are not statistically significant.

Positive juice samples contained fruit portions of apple, apricot, black currant, table grape, grapefruit, mango, orange, peach, pear, pineapple, or strawberry. Fruits not present in positive juice samples were aronia, banana, blueberry, sour cherry, cranberry, lemon, pomegranate, and raspberry.

In positive samples, ten active substances were found: boscalid, cyprodinil, flonicamid, fludioxonil, fluopyram, kresoxim-methyl, pirimicarb, pyrimethanil, tebuconazole, and trifloxystrobin. Four of these are PFASs: flonicamid, fludioxonil, fluopyram, and trifloxystrobin. The most frequently detected substances were fludioxonil, found in 13 samples (14.3% of all samples), and boscalid and fluopyram, each found in 10 samples (11.0% of all samples). Concentrations of active substances ranged from 0.005 to 0.065 mg/L. The results for concentrations in juice samples are presented in Table 2.

In Spain, 43% of analyzed fruit juice samples tested positive. Among the active substances, tebuconazole, which was analyzed but not found in the present study, was quantified at concentrations up to 0.017 mg/L [26]. In Poland, 60% of berry fruit juices (black currant, redcurrant, raspberry, strawberry) contained pesticide residues at concentrations up to 0.33 mg/kg. Among the active substances examined in the present study, boscalid (the most frequently found), cyprodinil, pyrimethanil, fludioxonil, and trifloxystrobin were found. These substances were also present in samples from this survey [27]. In Serbia, 71.2% of analyzed fruit juices were positive, and 60.0% contained multiple residues, which is higher than in the present survey. The highest number of active substances per sample was six, and the highest concentration was 0.629 mg/kg, which is also higher than in Slovenia. Among the active substances analyzed in the present study, azoxystrobin, cyprodinil, myclobutanil, pyrimethanil, pyriproxyfen, and trifloxystrobin were found. Of these, cyprodinil, pyrimethanil, and trifloxystrobin were also present in juices in Slovenia [28]. In Turkey, 98.2% of pomegranate juices tested positive for pesticide residues, with 45.0% containing multiple residues. Among the substances analyzed in the present survey, azoxystrobin, boscalid, and fludioxonil were quantified at concentrations up to 0.014 mg/kg. The latter two active substances were also found in the study in Slovenia [32]. The present study included only one pomegranate juice sample, for which none of the targeted pesticides were above the analytical threshold.

A limitation of this study is that residues in fruit juices were monitored only for one month in 2026. The proportion of positive samples was lower in the present study (29.7%) than in the 2025 study (42.5%) [34]. The purpose of the present research is to provide information about pesticide residues, including PFASs, in processed food. This information on food safety will allow data scientists to recommend which types of juices need to be inspected.

### 3.2. Calculated Estimated Residues in Fruit

Calculations of the estimated residues in fruit revealed compliance with MRLs, with the exception of four fruit samples containing fluopyram, representing possible exceedances in mango (sample designation 26-628) and table grape (sample designations 26-738, 26-746, 26-748). Possible exceedances were also observed for kresoxim-methyl in apple (sample designation 26-623), tebuconazole in mango (sample designation 26-734), and trifloxystrobin in apple (sample designations 26-623, 26-746). Altogether, six samples had possible MRL exceedances in their fruit: sample 26-623 had possible exceedances of kresoxim-methyl and trifloxystrobin in apple, and sample 26-746 had two possible exceedances of fluopyram and trifloxystrobin in table grape and apple, respectively. Samples with possible exceedances originated from Austria (one sample), Italy (one sample), and Slovenia (four samples). This represented 5.9% of samples from foreign countries (2 out of 34 samples) and 7.0% of samples from Slovenia (4 out of 57 samples). The calculated reconstructed concentrations in fruit are presented in Table 3.

When calculation refinement by alternative scenario was conducted to estimate residues in fruit by assuming that all residue in a juice sample was equally distributed among the portions of individual fruits, only one fruit sample (sample 26-628) showed an estimated MRL exceedance, indicating probable MRL exceedance of fluopyram in mango. After refinement, only 1.1% of samples possibly exceeded their MRLs. This approach cannot be treated as a reliable estimate of MRL compliance, as the residue may originate entirely from a single fruit ingredient. The refinement results by alternative scenario of possible MRL exceedances are presented in Table 4, and the refinement results by alternative scenario of the calculated estimated concentrations in apple for boscalid, fludioxonil, and tebuconazole, which can potentially lead to acute exposure, are presented in Table 5.

In Serbia, 2.2% of samples exceeded the MRLs in fruit [26], while in the present study, the percentage was 6.6% under the first scenario (the entire residue in the juice sample belongs to each type of fruit) and 1.1% under the alternative scenario (all residue in a juice sample was evenly distributed among the sum of portions of the individual fruits).

A limitation of this approach of back-calculating from juice to fruit is the uncertainty of the calculated concentration results. Although PFs are usually <1, when a PF of 1 was used, underestimation of concentrations in fruit could occur, and possible MRL exceedances could be overlooked. However, the same approach using PFs is adopted by EFSA in its annual reports on the monitoring of pesticide residues in food. This approach is also used by laboratories conducting official control of food on the market. When processed fruit is analyzed, laboratories have to conduct a risk assessment to decide whether the product can remain on the market or has to be withdrawn from it. Despite the uncertainty associated with the back-calculation approach, at the moment, this is the best way to determine what remains on or what is removed from the market.

### 3.3. Risk Assessment

Estimated daily intakes (EDIs) for chronic exposure and Estimated Short-Term Intakes (ESTIs) for acute exposure were calculated using the EFSA PRIMO model and are expressed as a percentage of the ADI and ARfD, respectively. The diets with the highest EDIs were DE child for boscalid, fludioxonil, kresoxim-methyl, pirimicarb, tebuconazole, and trifloxystrobin, and NL toddler for cyprodinil, flonicamid, fluopyram, and pyrimethanil.

Calculations of acute exposure show that there is a possible acute risk for boscalid in apples consumed by children (480% ARfD) and adults (125% ARfD), and in apple juice consumed by children (107% ARfD), but not by adults. A possible acute risk was also observed for fludioxonil (fluorinated pesticide) in apples consumed by children (110% ARfD), but not by adults, and not in apple juice consumed by children or adults. Finally, a possible acute risk was found for tebuconazole in apples consumed by children (101% ARfD), but not in apples consumed by adults or in juice consumed by children or adults. The acute risk assessment in EFSA PRIMo Rev 3.1, using input values only for raw products (for instance, apples), automatically calculates exposure for both raw and processed fruit (in the case of apples, apple juice). Data on possible exceedances in apple juice were not calculated directly from measurements of concentrations in apple juice.

The results show that fruits in which potential MRL exceedances were observed (fluopyram in table grape, fluopyram and tebuconazole in mango, kresoxim-methyl and trifloxystrobin in apple) do not pose a risk to consumers. This can be explained by the fact that MRLs are trade standards and not toxicological limits. Theoretical acute risk was posed by apple, where the maximum concentrations of boscalid, fludioxonil, and tebuconazole were estimated to be close to the MRLs but did not exceed them.

The EFSA PRIMo model rev. 3.1 automatically uses the ADI as the input value for calculations of acute exposure when the ARfD has not been set. This is because the ARfD value is always higher than—or, in the worst case, equal to—the ADI. It should be noted that, during the authorization process for PPPs and active substances, acute exposure is not calculated when an ARfD is not set, as in the case of boscalid and fludioxonil, since it is assumed that such substances are not acutely toxic. Therefore, it can be stated with certainty that theoretical acute risk is observed only for tebuconazole. The results are presented in Table 6, while detailed calculations of chronic exposure are presented in Tables S3–S13 in the Supplementary Materials.

The refinement of acute exposure by alternative approach was conducted by calculating estimated residues in fruit, assuming that all residue in a juice sample was evenly distributed among the sum of portions of the individual fruits (for example, for a juice sample containing 20% apples and 10% peach, the fruit portion is 30%). Since the ESTI was potentially exceeded for boscalid, fludioxonil, and tebuconazole only in apples consumed by children, refinement by alternative approach was conducted for apples only (Table 7). For apples, data from Table 5 were used in the calculations, and only boscalid potentially exceeded 100% of the ARfD for apples consumed by children (154% of the ARfD). It should be noted that no ARfD was allocated for boscalid and that using the ADI value instead of the ARfD probably results in an overestimation. Therefore, based on alternative approach calculations, it can be concluded with certainty only that, theoretically, no active substance posed an acute risk to consumers.

As in the present research, previous research has shown that chronic exposure to analyzed fruit juices from Serbia, Poland, and Turkey does not pose a risk to consumers [27,28,32]. In contrast to the present study, boscalid and fludioxonil were not analyzed in Serbia, and tebuconazole was not found there. However, the active substance carbofuran, which is no longer approved in the EU and was not included in the present study, posed an acute risk for preschool children in Serbia [28].

## 4. Conclusions

In the present study, 29.7% of fruit juices tested positive for at least one of the 32 targeted substances. After using the residues in juice to estimate the residues in fruit, assuming that all residues in each juice sample belonged to a type of fruit from which the juice was produced, and comparing these values with valid MRLs, 6.6% of samples potentially exceeded their MRLs. After recalculation using an alternative approach, assuming that all residues in each juice sample belonged to the whole fruit portion when the juice was produced from two or more types of fruit, only 1.1% of fruit samples potentially exceeded their MRLs. Multiple residues were present in 17.6% of juice samples, with ten active substances identified: boscalid, cyprodinil, flonicamid, fludioxonil, fluopyram, kresoxim-methyl, pirimicarb, pyrimethanil, tebuconazole, and trifloxystrobin. Four of these are PFASs: flonicamid, fludioxonil, fluopyram, and trifloxystrobin. While chronic exposure was acceptable, acute exposure was potentially unacceptable for tebuconazole in apple. After refining the calculation using an alternative approach, assuming that all residues in the juice sample belonged to the whole fruit portion from which the juice was produced, acute exposure was potentially acceptable.

In the future, this kind of research will continue to ensure the safety of processed food on the market.

## Figures and Tables

**Table 1:** Samples of fruit juices.

Sample Designation	Volume (L)	Content	Origin
26-590	1	orange 12%, lemon 1%	Austria
26-591	1	orange 25%	Austria
26-592	1	orange 100%	Austria
26-593	1	orange 100%	Austria
26-594	1	grapefruit 30%	Austria
26-595	1	apple 50%	Austria
26-596	1	apple 100%	Austria
26-597	1	peach 35%	Austria
26-598	1	strawberry 10%, apple 15%	Austria
26-599	1	blueberry 5%, apple 20%	Austria
26-600	1	black currant 20%	Austria
26-601	1	cranberry 25%, apple 5%	Austria
26-602	1	pineapple 100%	Austria
26-603	1	orange 50%	Slovenia
26-604	1	pineapple 100%	Slovenia
26-605	1	sour cherry 25%	Slovenia
26-606	1	apple 50%	Slovenia
26-607	1	lemon 6%, raspberry, strawberry, black currant, sour cherry 0.6%	Slovenia
26-608	1	strawberry 23%, apple 21%	Slovenia
26-609	1	peach 33%, apple 17%	Slovenia
26-610	1	apricot 25%, orange 9%, apple 8%	Slovenia
26-611	1	lemon 75%	Slovenia
26-612	1	mango 10%, peach 5%, orange 5%	Slovenia
26-613	1	pomegranate 25%	Slovenia
26-614	1	peach 25%	Slovenia
26-615	1	orange 100%	Hungary
26-616	1	orange 100%	Austria
26-617	1	orange 25%	Austria
26-618	1	orange 100%	Italy
26-619	1	apple 100%	Slovenia
26-620	1	apple 100%	Austria
26-621	1	apple 100%	Italy
26-622	1	strawberry 23%, apple 22%	Slovenia
26-623	1	strawberry 30%, apple and black currant 10%	Austria
26-624	1	pineapple 100%	Italy
26-625	1	pineapple 100%	Italy
26-626	1	apricot 40%	Austria
26-627	1	peach 25%	Austria
26-628	1	peach 32%, mango 10%	Italy
26-629	1	blueberry 11%, apple 9%	Austria
26-708	1	peach 100%	Slovenia
26-709	1	apple 100%	Slovenia
26-710	1	pear 100%	Slovenia
26-711	1	orange 100%	Slovenia
26-712	1	grape 100%	Slovenia
26-713	1	apple 100%	Slovenia
26-714	1	aronia 100%	Slovenia
26-715	1	orange 100%	Slovenia
26-716	1	pineapple 100%	Slovenia
26-717	1	strawberry 25%, apple 18%, grape 2%	Slovenia
26-718	1	apple 100%	Slovenia
26-719	1	apple 33%, aronia 10%, grape 7%	Slovenia
26-720	1	apricot 25%	Slovenia
26-721	1	peach 25%	Slovenia
26-722	1	apple 50%	Slovenia
26-723	1	grape 17%, blueberry 3%	Slovenia
26-724	1	apple 50%	Slovenia
26-725	1	apple 28.1%, grape 15%, currant 2.8%, sour cherry 2.3%, strawberry 1%, raspberry 0.8%	Slovenia
26-726	1	apple 100%	Slovenia
26-727	1	apple 7%, raspberry 3%	Slovenia
26-728	1	pineapple 100%	Slovenia
26-729	1	apple 100%	Slovenia
26-730	1	apricot 30%, apple 13%	Slovenia
26-731	1	pineapple 45%, orange 40%	Slovenia
26-732	1	peach 50%	Slovenia
26-733	1	apple 100%	Slovenia
26-734	1	peach 25%, apple 25%, grape 20%, mango 10%	Slovenia
26-735	1	apple 73%, grape 17%, lemon 3.7%, black currant 1.5%, aronia 1.3%, sour cherry 1%, raspberry 0.3%	Slovenia
26-736	1	orange 50%	Slovenia
26-737	1	strawberry 8%, apple 70%, aronia 5%	Slovenia
26-738	1	strawberry 30%, apple 11%, grape 4%	Slovenia
26-739	1	orange 100%	Slovenia
26-740	1	black currant 25%	Slovenia
26-741	1	apple 29%, pear 20%, peach 1%	Slovenia
26-742	0.2	apple 100%	Slovenia
26-743	0.2	peach 32%, apple 18%	Slovenia
26-744	0.2	black currant 25%	Slovenia
26-745	0.2	orange 100%	Slovenia
26-746	0.2	strawberry 30%, apple 11%, grape 4%	Slovenia
26-747	0.2	apple 100%	Serbia
26-748	0.2	strawberry 25%, apple 18%, grape 2%	Slovenia
26-749	0.2	orange 100%	Serbia
26-750	0.2	pineapple 100%	Slovenia
26-751	0.2	orange 11%	Austria
26-752	0.2	peach 7%, apple 5%	Austria
26-753	0.2	apple 12%	Austria
26-754	0.2	sour cherry 5%, orange 5%, banana 2%	Austria
26-755	0.2	apple 10%	Slovenia
26-756	0.2	strawberry 10%, apple 15%	Slovenia
26-757	0.2	apple 50%	Croatia
26-758	0.2	orange 50%	Croatia

**Table 2:** Results for positive fruit juice samples in mg/L.

Sample Designation	Boscalid	Cyprodinil	Flonicamid	Fludioxonil	Fluopyram	Kresoxim- methyl	Pirimicarb	Pyrimethanil	Tebuconazole	Trifloxystrobin
26-592				0.008				0.009		
26-594					0.024					
26-609	0.020			0.049	0.006					
26-610				0.009						
26-612				0.011						
26-618								0.009		
26-623	0.005				0.013	0.008				0.013
26-626	0.015				0.005				0.005	
26-627	0.006			0.011						
26-628	0.006			0.038	0.007				0.006	
26-708 *	0.021			0.065	0.007			0.008	0.008	
26-709								0.011		
26-710		0.010	0.005					0.029	0.010	
26-711								0.036		
26-717				0.011						
26-720	0.006									
26-721				0.011						
26-729			0.005				0.005			
26-730	0.016								0.012	
26-731				0.005						
26-732		0.005		0.015					0.005	
26-733							0.019			
26-734	0.006								0.012	
26-738				0.008	0.007					
26-743	0.010				0.005					
26-746					0.007					0.007
26-748				0.005	0.005					0.006

* To measure the concentration of fludioxonil, the sample was diluted fourfold before analysis.

**Table 3:** Calculated reconstructed concentrations in fruit in mg/kg.

	Apple	Apricot	Black Currant	Grape (Table)	GrapeFruit	Mango	Orange	Peach	Pear	Pineapple	Strawberry
					boscalid						
MRL	2	5	15	5		2		5			6
min	0.37	0.02	0.05	0.08		0.06		0.02			0.02
max	1.8	0.05	0.05	0.08		0.06		0.06			0.02
median	0.79	0.04	0.05	0.08		0.06		0.03			0.02
					cyprodinil						
MRL	2							2	2		
min	0.005							0.009	0.01		
max	0.005							0.009	0.01		
median	0.005							0.009	0.01		
					flonicamid						
MRL	0.3								0.3		
min	0.005								0.005		
max	0.005								0.005		
median	0.005								0.005		
				fludioxonil							
MRL	5	5		5		2	10	10		7	4
min	0.86	0.04		0.20		0.11	0.01	0.03		0.01	0.02
max	3.8	0.04		0.56		0.38	0.22	0.22		0.01	0.04
median	2.1	0.04		0.23		0.25	0.06	0.06		0.01	0.03
					fluopyram						
MRL	0.8	1.5	4	2	0.5	0.01		1.5			2
min	0.12	0.01	0.13	2.5	0.08	0.07		0.01			0.02
max	0.57	0.01	0.13	3.5	0.08	0.07		0.02			0.04
median	0.22	0.01	0.13	2.7	0.08	0.07		0.02			0.02
					kresoxim-methyl						
MRL	0.2		0.90								1.5
min	0.76		0.08								0.03
max	0.76		0.08								0.03
median	0.76		0.08								0.03
					pirimicarb						
MRL	0.5										
min	0.005										
max	0.02										
median	0.01										
					pyrimethanil						
MRL	15						8	10	15		
min	0.01						0.009	0.008	0.03		
max	0.01						0.04	0.008	0.03		
median	0.01						0.009	0.008	0.03		
					tebuconazole						
MRL	0.3	0.6		0.5		0.1		0.6	0.3		
min	0.14	0.01		0.06		0.06		0.01	0.01		
max	0.28	0.04		0.06		0.12		0.05	0.01		
median	0.21	0.03		0.06		0.09		0.01	0.01		
					trifloxystrobin						
MRL	0.7		3	3							1
min	0.44		0.13	0.17							0.02
max	1.6		0.13	0.32							0.04
median	0.77		0.13	0.24							0.03

**Table 4:** Refinement of calculated concentrations in fruits that exceeded MRLs in Table 3 in mg/kg.

	Apple	Grape (Table)	Mango
		fluopyram	
MRL		2	0.01
min		0.16	0.02
max		0.22	0.02
median		0.22	0.02
		kresoxim-methyl	
MRL	0.2		
min	0.20		
max	0.20		
median	0.20		
		tebuconazole	
MRL			0.1
min			0.02
max			0.02
median			0.02
		trifloxystrobin	
MRL	0.7		
min	0.19		
max	0.41		
median	0.30		

**Table 5:** Refinement of calculated estimated concentrations of active substances in fruits, in mg/kg, for those exceeding an ESTI of 100% of the ARfD.

	Apple
	boscalid
MRL	2
min	0.11
max	0.57
median	0.29
	fludioxonil
MRL	5
min	0.37
max	3.27
median	0.71
	tebuconazole
MRL	0.3
min	0.05
max	0.08
median	0.07

**Table 6:** Risk assessment for fruit using EFSA PRIMO model.

	ADI	ARfD	EDI	ESTI
	(mg/kg bw/d)	(mg/kg bw)	(% ADI)	(% ARfD)
Boscalid	0.04	/	25	480
Cyprodinil	0.03	/	0.3	5
Flonicamid	0.025	0.025	0.3	3
Fludioxonil	0.37	/	7	110
Fluopyram	0.012	0.5	55	51
Kresoxim-methyl	0.4	/	2	20
Pirimicarb	0.035	0.1	0.4	2
Pyrimethanil	0.17	/	0.2	3
Tebuconazole	0.03	0.03	9	101
Trifloxystrobin	0.1	0.5	10	34

**Table 7:** Refinement of acute risk assessment using values from Table 5 using the EFSA PRIMo model.

	ADI	ARfD	ESTI
	(mg/kg bw/d)	(mg/kg bw)	(% ARfD)
Boscalid	0.04	/	154
Fludioxonil	0.37	/	95
Tebuconazole	0.03	0.03	31

## Data Availability

The original contributions presented in this study are included in the article/Supplementary Materials. Further inquiries can be directed to the corresponding author.

## Supplementary Materials

The following supporting information can be downloaded at: https://www.mdpi.com/article/10.3390/foods15193521/s1, Table S1: Limit of quantification, procedural recoveries, matrix effect on apple matrix. Table S2: Processing factors for production of juices from fruit. Table S3: Average consumption data in g/kg bw/day for populations with highest chronic exposure. Table S4: Theoretical maximum daily intake (TMDI) expressed as % ADI for 5 most critical diets for boscalid. Table S5: Theoretical maximum daily intake (TMDI) expressed as % ADI for 5 most critical diets for cyprodinil. Table S6: Theoretical maximum daily intake (TMDI) expressed as % ADI for 5 most critical diets for flonicamid. Table S7: Theoretical maximum daily intake (TMDI) expressed as % ADI for 5 most critical diets for fludioxonil. Table S8: Theoretical maximum daily intake (TMDI) expressed as % ADI for 5 most critical diets for fluopyram. Table S9: Theoretical maximum daily intake (TMDI) expressed as % ADI for 5 most critical diets for kresoxim-methyl. Table S10: Theoretical maximum daily intake (TMDI) expressed as % ADI for 5 most critical diets for pirimicarb. Table S11: Theoretical maximum daily intake (TMDI) expressed as % ADI for 5 most critical diets for pyrimethanil. Table S12: Theoretical maximum daily intake (TMDI) expressed as % ADI for 5 most critical diets for tebuconazole. Table S13: Theoretical maximum daily intake (TMDI) expressed as % ADI for 5 most critical diets for trifloxystrobin.

## Abbreviations

The following abbreviations are used in this manuscript:

ADI	Acceptable Daily Intake
ARfD	Acute Reference Dose
EDI	Estimated Daily Intake
EFSA	European Food Safety Authority
ESTI	Estimated Short-Term Intake
GC-MS/MS	Gas Chromatograph coupled with tandem Mass Spectrometer
LOQ	Limit of Quantification
MRL	Maximum Residue Level
PF	Processing Factor
PFASs	Per- and Polyfluoroalkyl Substances
PPP	Plant Protection Product
