# Supplementary material for: A Market Survey of Pesticide Residues in Fruit Juices in Slovenia in 2026
Source: Foods. 2026 Oct 1;15(19):3521. doi: 10.3390/foods15193521 (PMC13665386; doi:10.3390/foods15193521)
Supplement: Supplementary file 1 [file foods-15-03521-s001.zip › foods-4538326-supplementary.pdf]

Supplement

**Table S1.** Limit of quantification, **p**rocedural recoveries, matrix effect on apple matrix.

| Active substance | LOQ (mg $L^{-1}$) | Average recovery[a] (%) | RSD[b] (%) | ME[c] (%) |
|---|---|---|---|---|
| Azoxystrobin | 0.005 | 91.6 | 6.6 | 272 |
| Benthiavalicarb-isopropyl | 0.005 | 97.9 | 9.0 | 270 |
| Boscalid | 0.005 | 87.8 | 2.7 | 205 |
| Clomazone | 0.005 | 85.8 | 5.3 | 49 |
| Cyprodinil | 0.005 | 84.9 | 6.9 | 70 |
| Flonicamid | 0.005 | 78.9 | 12.6 | 79 |
| Fuazifop-p-butyl | 0.005 | 84.1 | 3.3 | 105 |
| Fudioxonil | 0.005 | 88.3 | 3.8 | 170 |
| Flufenacet | 0.005 | 88.9 | 2.3 | 111 |
| Fluopicolide | 0.005 | 86.5 | 3.4 | 159 |
| Fuopyram | 0.005 | 88.8 | 7.0 | 112 |
| Flutolanil | 0.005 | 86.0 | 10.7 | 158 |
| Iprovalicarb | 0.005 | 94.2 | 2.9 | 186 |
| Kresoxim-methyl | 0.005 | 88.0 | 3.0 | 85 |
| Lambda-cyhalothrin | 0.005 | 75.8 | 9.9 | 172 |
| Metazachlor | 0.005 | 86.0 | 5.6 | 75 |
| Metribuzin | 0.005 | 81.4 | 6.0 | 38 |
| Myclobutanil | 0.005 | 88.9 | 4.6 | 107 |
| Napropamide | 0.005 | 86.4 | 3.0 | 121 |
| Penconazole | 0.005 | 86.7 | 7.6 | 55 |
| Pendimethalin | 0.005 | 82.3 | 9.9 | 39 |
| Pirimicarb | 0.005 | 87.0 | 5.8 | 57 |
| Proquinazid | 0.005 | 84.8 | 6.8 | 121 |
| Prosulfocarb | 0.005 | 84.2 | 5.6 | 58 |
| Pyraclostrobin | 0.005 | 100.7 | 16.6 | 363 |
| Pyrimethanil | 0.005 | 86.9 | 4.6 | 57 |
| Pyriproxyfen | 0.005 | 82.2 | 3.3 | 147 |
| Tebuconazole | 0.005 | 89.9 | 4.9 | 216 |
| Tebufenpyrad | 0.005 | 81.6 | 3.5 | 156 |
| Tefluthrin | 0.005 | 74.9 | 5.4 | 48 |
| Tetraconazole | 0.005 | 87.2 | 8.4 | 85 |
| Trifloxystrobin | 0.005 | 82.8 | 3.1 | 182 |

[a] Average recovery from 6 spiked samples obtained during analyses

[b] RSD for recoveries of 6 spiked samples obtained during analyses

[c] ME = matrix effect

**Table S2.** Processing factors for production of juices from fruit [38].

| | Apple | Apricot | Black currant | Grape (table) | Grapefruit | Mango | Orange | Peach | Pear | Pineapple | Strawberry |
|---|---|---|---|---|---|---|---|---|---|---|---|
| Boscalid | 0.07 | | | 0.42 | | | | | | | |
| Cyprodinil | | | | 0.3 | | | | | | | |
| Flonicamid | | | | | | | | 0.89 | | | |
| Fludioxonil | 0.03 | | | | | | | | | | |
| Fluopyram | 0.23 | | | 0.07 | | | | | | | |
| Kresoxim-methyl | 0.1 | | | 0.19 | | | | | | | |
| Pyrimicarb | | | | | | | | | | | |
| Pyrimethanil | 0.51 | | | | | | | | | | |
| Tebuconazole | 0.33 | | | | | | | | | | |
| Trifloxystrobin | 0.08 | | | | | | | | | | |

**Table S3.** Average consumption data in g/kg bw/day for populations with highest chronic exposure.

| Commodity/Diet | DE child | NL toddler | DE general | DE women 14-50 y | GEMS/Food GO6 |
|---|---|---|---|---|---|
| Apple | 12.4800 | 10.7830 | 2.4262 | 2.5763 | 0.9247 |
| Apricot | 0.3800 | 0.2500 | 0.0959 | 0.1064 | 0.1133 |
| Black currant | 0.0900 | 0.3330 | 0.0409 | 0.0465 | 0.0017 |
| Grapefruit | 0.2200 | 0.0600 | 0.0866 | 0.0854 | 0.0495 |
| Mango | 0.0300 | 0.1520 | 0.0345 | 0.0504 | 0.1042 |
| Orange | 4.0200 | 2.2430 | 1.5629 | 1.9125 | 0.9985 |
| Peach | 0.2800 | 0.2720 | 0.1270 | 0.1463 | 0.2610 |
| Pear | 0.6500 | 4.3390 | 0.1222 | 0.1429 | 0.0890 |
| Pineapple | 0.1700 | 0.2360 | 0.0941 | 0.1104 | 0.0217 |
| Strawberry | 0.5000 | 0.3400 | 0.1090 | 0.1191 | 0.0422 |
| Table grapes | 1.3700 | 1.5480 | 0.2558 | 0.3013 | 1.0518 |

**Table S4.** Theoretical maximum daily intake (TMDI) expressed as % ADI for 5 most critical diets for boscalid.

| Commodity/Diet | DE child | NL toddler | DE general | DE women 14-50 y | GEMS/Food GO6 |
|---|---|---|---|---|---|
| Apple | 24.65 | 21.30 | 4.79 | 5.09 | 1.83 |
| Apricot | 0.04 | 0.03 | 0.01 | 0.01 | 0.01 |
| Black currant | 0.01 | 0.04 | 0.01 | 0.01 | 0.0002 |
| Mango | 0.005 | 0.02 | 0.01 | 0.01 | 0.02 |
| Peach | 0.02 | 0.02 | 0.01 | 0.01 | 0.02 |
| Strawberry | 0.03 | 0.02 | 0.01 | 0.01 | 0.002 |
| Table grapes | 0.27 | 0.31 | 0.05 | 0.06 | 0.21 |
| Sum | 25.03 | 21.74 | 4.89 | 5.20 | 2.09 |

**Table S5**. Theoretical maximum daily intake (TMDI) expressed as % ADI for 5 most critical diets for cyprodinil.

| Commodity/Diet | DE child | NL toddler | DE general | DE women 14-50 y | GEMS/Food GO6 |
|---|---|---|---|---|---|
| Apple | 0.21 | 0.18 | 0.04 | 0.04 | 0.02 |
| Peach | 0.01 | 0.01 | 0.004 | 0.004 | 0.01 |
| Pear | 0.02 | 0.14 | 0.004 | 0.005 | 0.003 |
| Sum | 0.24 | 0.33 | 0.05 | 0.05 | 0.03 |

**Table S6**. Theoretical maximum daily intake (TMDI) expressed as % ADI for 5 most critical diets for flonicamid.

| Commodity/Diet | DE child | NL toddler | DE general | DE women 14-50 y | GEMS/Food GO6 |
|---|---|---|---|---|---|
| Apple | 0.25 | 0.22 | 0.05 | 0.05 | 0.02 |
| Pear | 0.01 | 0.09 | 0.002 | 0.003 | 0.002 |
| Sum | 0.26 | 0.31 | 0.05 | 0.05 | 0.02 |

**Table S7**. Theoretical maximum daily intake (TMDI) expressed as % ADI for 5 most critical diets for fludioxonil.

| Commodity/Diet | DE child | NL toddler | DE general | DE women 14-50 y | GEMS/Food GO6 |
|---|---|---|---|---|---|
| Apple | 6.98 | 6.03 | 0.03 | 1.44 | 0.52 |
| Apricot | 0.004 | 0.003 | 0.001 | 0.001 | 0.001 |
| Mango | 0.002 | 0.01 | 0.002 | 0.003 | 0.01 |
| Orange | 0.07 | 0.04 | 0.03 | 0.03 | 0.02 |
| Peach | 0.005 | 0.004 | 0.002 | 0.002 | 0.004 |
| Pineapple | 0.0005 | 0.001 | 0.0003 | 0.0003 | 0.0001 |
| Strawberry | 0.004 | 0.003 | 0.001 | 0.001 | 0.0003 |
| Table grapes | 0.09 | 0.10 | 0.02 | 0.02 | 0.07 |
| Sum | 7.16 | 6.19 | 0.09 | 1.50 | 0.63 |

**Table S8**. Theoretical maximum daily intake (TMDI) expressed as % ADI for 5 most critical diets for fluopyram.

| Commodity/Diet | DE child | NL toddler | DE general | DE women 14-50 y | GEMS/Food GO6 |
|---|---|---|---|---|---|
| Apple | 22.88 | 19.77 | 4.45 | 4.72 | 1.70 |
| Apricot | 0.03 | 0.02 | 0.01 | 0.01 | 0.01 |
| Black currant | 0.10 | 0.36 | 0.04 | 0.05 | 0.002 |
| Grapefruit | 0.15 | 0.04 | 0.06 | 0.06 | 0.03 |
| Mango | 0.02 | 0.09 | 0.02 | 0.03 | 0.06 |
| Peach | 0.05 | 0.05 | 0.02 | 0.02 | 0.04 |
| Strawberry | 0.08 | 0.06 | 0.02 | 0.02 | 0.01 |
| Table grapes | 30.60 | 34.57 | 5.71 | 6.73 | 23.49 |
| Sum | 53.91 | 54.96 | 10.33 | 11.64 | 25.34 |

**Table S9**. Theoretical maximum daily intake (TMDI) expressed as % ADI for 5 most critical diets for kresoxim-methyl.

| Commodity/Diet | DE child | NL toddler | DE general | DE women 14-50 y | GEMS/Food GO6 |
|---|---|---|---|---|---|
| Apple | 2.37 | 2.05 | 0.46 | 0.49 | 0.18 |
| Black currant | 0.002 | 0.01 | 0.001 | 0.001 | 0.00003 |
| Strawberry | 0.004 | 0.003 | 0.001 | 0.001 | 0.0003 |
| Sum | 2.37 | 2.06 | 0.46 | 0.49 | 0.18 |

**Table S10**. Theoretical maximum daily intake (TMDI) expressed as % ADI for 5 most critical diets for pirimicarb.

| Commodity/Diet | DE child | NL toddler | DE general | DE women 14-50 y | GEMS/Food GO6 |
|---|---|---|---|---|---|
| Apple | 0.36 | 0.31 | 0.07 | 0.07 | 0.03 |

**Table S11**. Theoretical maximum daily intake (TMDI) expressed as % ADI for 5 most critical diets for pyrimethanil.

| Commodity/Diet | DE child | NL toddler | DE general | DE women 14-50 y | GEMS/Food GO6 |
|---|---|---|---|---|---|
| Apple | 0.07 | 0.06 | 0.01 | 0.02 | 0.01 |
| Orange | 0.02 | 0.01 | 0.01 | 0.01 | 0.01 |
| Peach | 0.001 | 0.001 | 0.001 | 0.001 | 0.001 |
| Pear | 0.01 | 0.08 | 0.002 | 0.003 | 0.002 |
| Sum | 0.10 | 0.15 | 0.02 | 0.03 | 0.02 |

**Table S12**. Theoretical maximum daily intake (TMDI) expressed as % ADI for 5 most critical diets for tebuconazole.

| Commodity/Diet | DE child | NL toddler | DE general | DE women 14-50 y | GEMS/Food GO6 |
|---|---|---|---|---|---|
| Apple | 8.74 | 7.55 | 1.70 | 1.80 | 0.65 |
| Apricot | 0.04 | 0.03 | 0.01 | 0.01 | 0.01 |
| Mango | 0.01 | 0.05 | 0.01 | 0.02 | 0.03 |
| Peach | 0.01 | 0.01 | 0.004 | 0.005 | 0.01 |
| Pear | 0.02 | 0.14 | 0.004 | 0.005 | 0.003 |
| Table grapes | 0.27 | 0.31 | 0.05 | 0.06 | 0.21 |
| Sum | 9.09 | 8.09 | 1.78 | 1.90 | 0.91 |

**Table S13**. Theoretical maximum daily intake (TMDI) expressed as % ADI for 5 most critical diets for trifloxystrobin.

| Commodity/Diet | DE child | NL toddler | DE general | DE women 14-50 y | GEMS/Food GO6 |
|---|---|---|---|---|---|
| Apple | 9.61 | 8.30 | 1.87 | 1.98 | 0.71 |
| Black currant | 0.01 | 0.04 | 0.01 | 0.01 | 0.0002 |
| Strawberry | 0.02 | 0.01 | 0.003 | 0.004 | 0.001 |
| Table grapes | 0.33 | 0.37 | 0.06 | 0.07 | 0.25 |
| Sum | 9.97 | 8.72 | 1.94 | 2.06 | 0.96 |
